# How brand attitude, brand quality, and brand value affect Thai canned tuna consumer brand loyalty

**DOI:** 10.1016/j.heliyon.2021.e06301

**Published:** 2021-02-23

**Authors:** Pichit Chuenban, Puris Sornsaruht, Paitoon Pimdee

**Affiliations:** a Faculty of Administration and Management (FAM), King Mongkut's Institute of Technology Ladkrabang (KMITL), Thailand; b KMITL Business School (KBS), King Mongkut's Institute of Technology Ladkrabang (KMITL), Bangkok, Thailand; c Faculty of Industrial Education and Technology, King Mongkut's Institute of Technology Ladkrabang (KMITL), Bangkok, Thailand

**Keywords:** Brand attitude, Brand quality, Brand value, Food security, Sustainability, Thailand

## Abstract

As one of the world's most valuable fish, Tuna can either be found in inexpensive tins on grocery store shelves or on high-end auction blocks destined for Japanese sashimi and sushi restaurants. Moreover, Thailand is reported to be the world's largest exporter of prepared or preserved tuna, which in 2019 contributed an estimated $2.5 billion to Thailand's exports. However, brand research is very limited for canned tuna, especially when it comes to product packaging cues, and how packaging affects a consumer's decision-making process. Therefore, this study set out to investigate which factors play the greatest role in a Thai consumer's brand loyalty (BL) for tuna fish canned products. Research teams using systematic random sampling were dispatched to five supermarkets within the Bangkok metropolitan area and collected 400 questionnaires answering items related to brand attitudes (BA), brand quality (BQ), and brand value (BV) as they related to canned tuna brand loyalty (BL). Thereafter, data analysis of the four constructs and 13 observed variables was conducted with use of LISREL 9.1, which included a confirmatory factor analysis (CFA), a goodness-of-fit (GOF) analysis along with the study's structural equation modeling (SEM). Results determined that there was a positive effect (73% R^2^) between all the causal factors on Thai canned tuna BL. Furthermore, the factors affecting BL ranked from the greatest to the lowest were BA, BQ, and BV with total effect (TE) values of 0.85, 0.33, and 0.19, respectively. Key takeaways from the study's research seem to indicate that canned tuna is viewed by most consumers as a ‘commodity’, with product availability and convenience being of the utmost importance in product brand selection. Global fishing stocks sustainability, labor costs/practices, and dolphin free catches within the tuna industry were also determined to be important aspects within the international consumer community. Also, due to the demand for high-quality and safe tuna products, adding value has become increasingly important in satisfying consumer demands and represents an opportunity for marketplace expansion.

## 1 Introduction

It has been stated that the story concerning tuna is without borders, which crosses millenniums of time, and wherever tuna was landed ashore, conflict was not far away (Adolf, 2019). The story of tuna is also a story of past limitless abundance to present day scarcity and exceptional monetary value (Ellis, 2008), which can also be an excellent case study for students of global sustainability and food security.

The famous French naval officer, oceanographer, and underwater explorer, Jacques Cousteau, was one of the first individuals to bring the underwater world in film to the masses and stated that “*you conserve what you love*” (Adolf, 2019, p.3) Later, Cousteau (2004) wrote about his harrowing *almadraba* tuna killing experiences with tuna fishermen at a tuna port near the ancient city of Carthage, near present day Tunisia. Simply stated, the *almadraba* is a three millennium old traditional tuna fishing technique (*tonnara*) for the trapping and slaughter of Atlantic/Northern Bluefin tuna (*Thunnus thynnus*), making their way from the Atlantic Ocean to the Mediterranean Sea from February to July to spawn (Ambrosio and Xandri, 2015). This Stone Age technique is mirrored across the Mediterranean and goes by other names such as *Mattanza* (the killing) and *Almadrava* (a place to strike) (Maggio, 2000). It has also been observed that the Mediterranean people viewed this ancient migration of the Bluefin tuna in much the same way America's native Indians viewed the hunting and killing of the buffalo.

Even today Bluefin tuna is a crucial species of fish in both value and employment source within the European Union (EU), and is of socio-economic and cultural relevance (Ambrosio and Xandri, 2015). Further support for tuna fishing's cultural importance can be found in the masterpiece from Salvador Dalí’s 1967 painting entitled *Tuna Fishing* (Ellis, 2008). Depicting the violent, chaotic battle of men in the *Almadrava* with huge tuna, a golden knife stabs into the tuna and the sea of azure blue turns blood red (Pavesic, 2011). Also, Almadraba fishing techniques are also more labor-intensive than any other fishing method whose production is increasingly appreciated by a high-end domestic and international consumer market.

However, in spite of being a consistent form of protein for Mediterranean Sea people, tuna fishing did not become a global industry until 1903, where in the United States industrial tuna fishing began as a potential replacement source for dwindling sardine catches (Butler, 2015), with the first canned tuna entering the market in the same year (Smith, 2012). Although in the beginning Americans turned their noses up at the canned product, by 1918 canned tuna had become the second most popular consumer seafood. Moreover, as the US entered WWII, canned tuna became an importance source of protein for the war, both domestically and for military personnel overseas. Today, it is recognized as a source of high protein, potential Omega-3 benefits, and a low-cost diet alternative (Chavan, 2020). However, in spite of these positives aspects, Majerová et al. (2013) in research from Slovakia has warned that consumers are driving trade enterprises to direct their efforts towards consumers' wishes and to be aware of their own responsibility for society's welfare.

After WWII, canned tuna continued to gain in popularity, with 100s of large tuna clipper ships plying the seas, dozens of tuna canneries along the US coasts, and numerous brands employing hundreds of thousands of workers (Smith, 2012). Moreover, by 1954, U.S. companies and consumers led the world market for canned tuna, with tuna continuing its popularity for decades to come. Finally, by 1990, the International Trade Commission (ITC) reported that Americans were eating up to two-thirds of the global supply of canned tuna (Ferdman, 2014).

However, it was also in 1990 when American consumers began to lose their loyalty to canned tuna, with a variety of factors coming into play. These included concerns about the product's susceptibility to mercury poisoning, killing of dolphins from tuna nets, overconsumption, and higher prices (Ferdman, 2014). However, in an eight year study from 2009 – 2016, Ormaza-González et al. (2020) determined that although there were low levels of cadmium, lead, and mercury discovered in tuna canned in Ecuadorian canneries, all levels were well below the maximum concentrations allowed by the reported literature.

In Asia, on January 30^th^, 1985, Thailand's Ministry of Commerce introduced measures for the export of tuna. In later research on these Thai tuna processing and exporting firms, Kuldilok (2009) stated that Thai tuna processing firms operate through price leadership by a dominant firm, with branding strategy regulated to canned products only. Moreover, although Thailand has maintained a comparative advantage within the global industry for many years, this is critically dependent on low labor costs, which according to Kuldilok (2009) is not consistent with Thailand's continued economic growth.

Moving forward to 2019, Thailand's tuna processors have risen to become the world's largest exporter of prepared or preserved tuna (Hasnan, 2019). Furthermore, of the estimated **$**25.63 billion global canned seafood industry in 2019, canned tuna from Thailand represented $2.2 billion in export value in 2018 (MICE Intelligence Center, 2019), with the primary export countries being the US, Australia, Japan, and Canada (Hasnan, 2019). National economic importance of tuna in the Asia-Pacific region is not just limited to Thailand either. Further out in the Western and Central Pacific, the Nauru Agreement has underscored tuna's importance as a primary natural resource to eight island nations. It is a David and Goliath story of how these tiny nations took on the massive (and sometimes unregulated) fishing fleets of China (Riskas, 2020), Japan, Korea, and the USA, forcing them to pay for access to their waters and to abide by the rules and regulations they have set to maintain the health of their tuna stock (Quince and Phillips, 2020).

Furthermore, domestically, Thailand like many other countries is also a large consumer of canned tuna, as it has been a mother's ‘go to’ choice for her family for generations. However, brand research is very limited for canned tuna, especially when it comes to product packaging cues, and how packaging affects a consumer's decision-making process (Midttun, 2015). Also, due to the demand for high-quality and safe tuna products, Morrissey and DeWitt (2013) stated that adding value has become increasingly important in satisfying consumer demands, and represents an opportunity for marketplace expansion. This is consistent with ground breaking low-income consumer (LIC) from Latin American research in which Prahalad (2004) and Prahalad and Hart (2010) wrote that once seen as price-driven, LICs are now brand-conscious consumers who are willing to pay a premium for quality. In Brazil, Filho et al. (2020) made similar determinations.

## 2 Literature review

### 2.1 Brand attitude (BA)

Wang (2013) also contributed to the research about consumer BA, and concluded that visual packaging affects a consumer's food product quality perception and their brand preference. This was consistent with O'Callaghan and Kerry (2016) who determined that as familiarity with a product increased, the consumer's attitude and acceptance of technologically related changes to the packaging increased. Grinsven and Das (2016) also studied the importance of brand logos, and suggested that brand logos that are simple offer benefits that are short-term, while complex logos give longer term benefits. According to Venter de Villiers et al. (2018), with female South African consumers, BA is often times used as a measure in determining a potential buyer's behavior. Yoon and Park (2012) have also added that BA is the perception of direction and strength of a consumer in relation to a brand. A consumer's BA also plays a significant role in the selection process as their association with a particular brand is an essential aspect in the success of a brand, since brands convey the consumer's attitude developed toward a brand (Ansary and Hashim, 2018). This is also consistent with research on Italian organic food consumers, in which Nosi et al. (2020) indicated the importance of a vendor's CSR image and the perceived value of their ecological welfare in a consumer's attitude. Therefore, the following three hypotheses were conceptualized for the study:

Hypothesis 1 The presence of a consumer's brand attitude positively and directly affects their perception of brand quality.

Hypothesis 2 The presence of a consumer's brand attitude positively and directly affects the brand's value.

Hypothesis 3 The presence of a consumer's brand attitude positively and directly affects the consumer's brand loyalty.

### 2.2 Brand quality (BQ)

Within the fishing industry, BQ has a long history. One example of this can be found in the Dutch/British herring wars of the 18^th^ century in which Scottish herring was branded and subsidized by the British for export to Europe (Webster, 2015). During this period, British branded herring won out over the similar Dutch products due to lower prices and acceptable quality. In a more contemporary way, Iceland borrowed earlier concepts of using national themes for BQ and developed an Icelandic logo for high quality fish products (Luten et al., 2003), which also has a clear environmental agenda strengthening the brand's image of quality. This is consistent with Calvo-Porral and Lévy-Mangin (2017) which reported that a consumer's perceived quality and to a lesser extent price, strongly affects their purchase intention. Therefore, the following two hypotheses were conceptualized for the study:

Hypothesis 4 Brand quality consumer perception positively and directly affects a brand's value.

Hypothesis 5 Brand quality consumer perception positively and directly affects the consumer's brand loyalty.

### 2.3 Brand value (BV)

Ryu et al. (2012) reported that brand value (BV) perception by consumers plays a crucial role in consumer satisfaction. Furthermore, within the canned tuna industry, Morrissey and DeWitt (2013) added that adding value to tuna products has become essential in for safe and high-quality tuna around the world. Specifics for adding value include longer shelf life, safer products, and enhancing the producer's economic return. Also, new product development and improved safety create a synergy which enhances success and competitiveness. Other BV attributes include sustainability and eco-labeling, as well as delivering new products which are healthy (Morrissey and DeWitt, 2013). Ansary and Hashim (2018) have also suggested that BV in the form of brand equity is driven by brand attachment, brand awareness, and brand attitude, which contribute to the relationship between brand equity and brand image. Within the restaurant industry, Ryu et al. (2012) also believed that the restaurant's physical environment and food quality determined a diner's perceived value. Therefore, the following hypothesis was conceptualized for the study:

Hypothesis 6 A consumer's perception of brand value positively and directly affects the consumer's brand loyalty.

### 2.4 Brand loyalty (BL)

According to Kaur et al. (2018), consumer behavior science studies state BL is dependent on brand affect and consumer satisfaction. Zollo et al. (2020) have added that in Italy, cognitive, social integrative, and personal integrative benefits determine BL. Additionally, Park (2009) reported that customer satisfaction and trust were essential in BL. This is consistent with Yoo et al. (2000) which determined that BL plays a critical role in a firm's competitiveness, sustainability and profitability, with strong brand association leading to greater BL.

Moreover, in Spain, Delgado-Ballester and Munuera-Aleman (2001) added that BL has a strong effect on a consumer's decision to purchase and inhibits their desire to move to a competitor's brand, while Han et al. (2015) reported on the positive influence of brand association on a brand's reputation. BL is at the core of BV.

Agrawal (2017) also added that consumers are starving for product information; with 56% of the consumers surveyed claiming brands inspire more trust by giving more product information. Therefore, organizations need to engage their customers, tell their stories, and share the unique value their products offer. Lee and Chang (2014) also added the powerful idea that within the tuna marketing industry, the tuna brand can be an organization's greatest asset. In Vietnam, Nguyen and Thanh (2016) added that a brand personality had a significant influence on BL within the frozen tuna market, with Viettrack (2009) reporting that 80% (*n = 600*) of their survey's Vietnamese consumers seldom switched tuna brands. In China, the *Guangdong Association of Distant-Water Fishing* when discussing the expansion of its fleet and tuna processing and trading center in November 2020 to feed national and regional demand, noted the importance of “building a national tuna brand” (Godfrey, 2020).

Therefore, from the authors' review, the factors determined to potentially play an important role in BL included the constructs BA, BQ, and BV along with their 13 related observed variables detailed in Table 1.

**Table 1:** Questionnaire constructs, their observed variables, and related theory support.

Constructs	Observed variables (46 questionnaire items)	Supporting theory
Brand Attitude (BA)	Canned tuna labelling (6 items) Canned tuna brand confidence (4 items) Canned tuna packaging (4 items) Canned tuna size and material (5 items)	(Andrews and Siengthai, 2009; Ansary and Hashim, 2018; Chaipraditkul, 2013; Chemerinski, 2012; Grinsven and Das, 2016; Hana, 2012; O'Callaghan and Kerry, 2016; Nosi et al., 2020; Salehzadeh and Pool, 2017; Wang, 2013; Zaeema and Hassan, 2016)
Brand Quality (BQ)	Canned tuna quality (3 items) Canned tuna taste (2 items) Canned tuna packaging quality (3 items)	(Calvo-Porral and Lévy-Mangin, 2017; Conte et al., 2014; Luten et al., 2003; Webster, 2015)
Brand Value (BV)	Canned tuna pricing (2 items) Canned tuna value (3 items) Canned tuna brand (2 items)	(Ansary and Hashim, 2018; Filho et al., 2020; Godfrey, 2020; Morrissey and DeWitt, 2013; Prahalad, 2004; Prahalad and Hart. 2010; Ryu et al., 2012)
Brand Loyalty (BL)	Famous brand (4 items) Brand loyalty (5 items) Brand quality (3 items)	(Agrawal, 2017; Baalbaki, 2012; Chow and Holden, 1997; Delgado-Ballester and Munuera-Aleman, 2001; Greenpeace USA, 2016; Han et al., 2015; Havice and Campling, 2018; Kaur et al., 2018; Lee and Chang, 2014; Nevin, 2003; Nguyen and Thanh, 2016; Park, 2009; Rust et al., 2000; Thomson, 2017; Viettrack, 2009; Yoo et al., 2000; Zollo et al., 2020)

### 2.5 Research objectives

1. To investigate the interrelationships through SEM of the factors influencing a consumer's brand loyalty (BL) to a particular canned tuna brand.
2. To conduct a GOF and CFA to confirm the model's fit before the SEM.
3. To make recommendations to vendors and entrepreneurs on which aspects lead to BL and, therefore, their increased profitability.

## 3 Materials and methods

The Human Ethics Committee at the King Mongkut's Institute of Technology Ladkrabang (KMITL) was consulted and approval was obtained prior to the meeting with experts concerning the questionnaire's design. An informed consent form for each of the study's pilot-survey group and main study's respondents were also obtained (Castro-Jiménez, 2020), with each participants anonymity considered and ensured. Reliability and validity assurance tools included the use of a CFA (Table 2) and GOF assessment before the model's six hypotheses SEM (Table 5) (Sitabutr and Pimdee, 2017). The methods described are in detail sufficient to understand the approach used and appropriate statistical tests which are applied.

**Table 2:** CFA construct reliability and validity.

Constructs	CR	AVE	α	Observed variables	loading	R^2^
Brand Quality (BQ)	0.893	0.699	0.920	Canned tuna quality (y4)	0.851	0.724
				Canned tuna taste (y5)	0.817	0.667
				Canned tuna packaging quality (y6)	0.840	0.706
Brand Value (BV)	0.851	0.657	0.905	Canned tuna pricing (y7)	0.800	0.639
				Canned tuna value (y8)	0.860	0.739
				Canned tuna brand (y9)	0.769	0.592
Brand Loyalty (BL)	0.894	0.741	0.925	Famous brand (y1)	0.951	0.905
				Brand loyalty (y2)	0.907	0.823
				Brand quality (y3)	0.704	0.496
Brand Attitude (BA)	0.883	0.635	0.924	Canned tuna labeling (x1)	0.771	0.594
				Canned tuna brand confidence (x2)	0.816	0.666
				Canned tuna packaging (x3)	0.764	0.584
				Canned tuna size and material (x4)	0.877	0.770

### 3.1 Population and sample size

The population consisted of retail supermarket shoppers from five local communities within the Bangkok metropolitan area. Sample size for the study was determined from theory in which various scholars have suggested that sample sizes from 200 - 400 individuals depending on model complexity and the number of variables (Hair et al., 2016). Moreover, Mertler (2016) has suggested that in education research for population sizes of approximately 1,500, a sample of 300 is sufficient. Therefore, in order to maintain stringent standards for the survey, a sample of 400 was determined, which was obtained after the audit's completion (Kyriazos, 2018).

### 3.2 Data collection

Graduate student research teams were dispatched to five supermarkets in the Bangkok metropolitan area communities of Pathumwan, Bangkapi, Bangkae, Bangna, and Ladprao. By use of systematic random sampling, every fifth shopper was asked to participate in the Thai university study concerning their opinions about canned tuna products (Crawford, 1997; Mohamad and Ghani, 2014).

### 3.3 The design of the questionnaire

Each consumer's questionnaire consisted of two sections; with section 1 containing seven items about the consumer themselves. Section 2 consisted of four latent variables and 46 survey items (Table 1), with the seven-level agreement scale using ‘1’ as *strongly disagree* (0.00–1.84) and ‘7’ as *strongly agree* (6.14–7.00) as anchor points (Kaewnuch, 2019).

### 3.4 Research instrument qualtiy assessment

After the questionnaire's design, assessment of questionnaire's *content validity* (CV) was undertaken. According to Kelly (1999), a research study's design strength is strongly dependent on how accurately the variables which have been selected are measured, which is commonly referred to as *validity*. Moreover, validity denotes the extent to which specific items on a tool accurately assess the concept being measured in the study, and assures that the items being used allow valid inferences to be made. The three types of validity related to data measurement are 1) content (and face), 2) criterion (predictive), and 3) construct (Price et al., 2019). Therefore, the CV assessment process for the questionnaire was undertaken with the assistance of five experts in the fields of branding, statistics, and environmental studies. Each item was then accessed by use of an indexes of item-objective congruence (IOC) rating (Turner and Carlson, 2003) a) its relevance to the questionnaire's aim and b) its clarity. After which, each response was reviewed for its c) comprehensiveness and completeness and d) its meaningfulness and significance for each item.

The minimum IOC value for this study was 0.67. Items with a value less than 0.67 were removed or re-written according to the experts' suggestions (Pimdee, 2020). The reliability of the questionnaire was then evaluated using 35 students from local (campus and off-campus) university markets which stocked the canned tuna brands being surveyed. Assessment of the 35 individual pilot-test questionnaires' reliability used Cronbach's alpha (α) for the canned tuna survey items (Zaeema and Hassan, 2016).

However, in use of Cronbach's α, there is great controversy and misconceptions concerning the use of cut-off scores. A common misconception and common citation is for a α reliability of ≥.70 (Nunnally, 1978). However, this is not the case, and higher scores are actually suggested (Lance et al., 2006). Therefore, the authors raised the commonly accepted ≥.70 to ≥.80, as a better indicator of the research instrument's satisfactory level of reliability. In Table 1, we note that the α for the latent variables was from 0.905 to 0.925, indicating a higher level of item reliability.

## 4 Results

### 4.1 Questionnaire respondents' results (n = 400)

Survey results indicated that 59% were women, with 51.25% from 21 - 30 years old. Moreover, 64% were single with 89% having incomes less than 30,000 baht per month ($992.00 on 24 December 2020). Furthermore, 59.25% had obtained an undergraduate degree or higher. Somewhat interesting was 26.25% indicated they were entrepreneurs while 31.5% stated they were basic laborers, while another 19.25% reported they were a worker in a private company. Finally, a majority of 38.25% preferred the *Three Lady Cooks* canned tuna brand, 19% liked *Select*, with *Roza*, *Super-C-Chef*, and *Pumpui* all even at 14.25%.

### 4.2 Questionnaire pre-testing and validity measurement

A questionnaire pre-test (pilot test) was conducted by use of 30 questionnaires given to consumers whose data was not used in the subsequent sample (Converse and Presser, 1986). Prior to this, however, questionnaire development was undertaken with the assistance of five experts in their related fields, whose results were analyzed by use of the item-objective congruence (IOC). Pre-test results were analyzed by use of Cronbach's alpha (α), whose item reliability was judged to be excellent as the latent variable scores were 0.905–0.925 (Table 3) (Tavakol and Dennick, 2011).

**Table 3:** Mediation effects affecting BL.

Dependent variables		Independent variables			
		R^2^	BA	BQ	BV
BL	DE	.73	0.49∗∗	0.20	0.19
	IE		0.36∗	0.13	-
	TE		0.85∗∗	0.33∗	0.19
BV	DE	.85	0.32∗	0.67∗∗	-
	IE		0.60∗∗	-	-
	TE		0.92∗∗	0.67∗∗	-
BQ	DE	.79	0.89∗∗	-	-
	IE		-	-	-
	TE		0.89∗∗	-	-

∗*p* ≤ 0.05, ∗∗*p* ≤ 0.01.

### 4.3 CFA results

Prior to the structural equation modelling, a CFA was run (Table 2). Various software packages use different goodness-of-fit (GOF) indices but for LISREL 9.1 the most commonly used is the χ2/df with a criterion of ≤2.00 (Sahoo, 2019). Additional indices and their criteria used were CFI ≥0. .95, the GFI ≥0.90, *p* ≥ 0.05, and RMSEA ≤0.05 (Jöreskog et al., 2016), AGFI ≥0.90, and RMR ≤0.05, the SRMR ≤0.05, and the NFI ≥0.90 (Schumacker and Lomax, 2016). Furthermore, Hooper et al. (2008) has suggested that R^2^ values should not be lower than ≤0.20, while factor loadings should also be ≥0.5 and composite/construct reliability (CR) should also be ≥0.7. Thereafter, it is suggested that the model's fit validity should also be tested by use of an AVE ≥0.5. Therefore, from the GOF analysis, the CFA results showed that χ2/df = 0.81, the GFI = 0.99, the AGFI = 0.97, the SRMR = 0.01, the RMSEA = 0.00, and finally, α for the latent variables averaged 0.9185. Therefore, the model fit was determined to be good to excellent (Kaewnuch, 2019). Additionally, Netemeyer et al. (2003) have suggested that CFA reliability and internal consistency testing for CR should be ≥0.80. As CR values from the analysis were 0.851–0.894, this high criterion was met. Finally, Mustofa and Mulyono (2020) have also stated that loading factors and variable AVE values should be ≥0.5 for the variables to be reliable and valid.

### 4.4 Mediation effects

It was determined that the model's causal variables had a positive effect on BL, which when combined have an R^2^ of 73% on BL. Furthermore, total effect (TE) values of each construct when ranked in importance are BA, BQ, and BV, with TE = 0.85, TE = 0.33, and TE = 0.19, respectively (Table 3).

### 4.5 Correlation coefficient testing results

Hypotheses testing results from the SEM revealed four weak to strong correlations (Table 5) based on recommendations from Pearson's *r* of 0.10–0.29 as weak, 0.30 to 0.49 as moderate, and values form 0.50 to 1 as strong (Table 4) (Akoglu, 2018; Ratner, 2009). Hair et al. (2016) also added that construct validity could be judged acceptable when t-values ≥ 1.96. Sharma (1996) has also added that when standardized factor loading ≥0.60, further validity can be ascertained.

**Table 4:** The *r*, CR, and AVE of the latent variables (under the **bold** diagonal).

Constructs	BQ	BV	BL	BA
Brand Quality (BQ)	**1.00**			
Brand Value (BV)	0.959∗∗	**1.00**		
Brand Loyalty (BL)	0.828∗∗	0.843∗∗	**1.00**	
Brand Attitude (BA)	0.891∗∗	0.921∗∗	0.853∗∗	**1.00**
Construct/Composite Reliability	0.893∗∗	0.851∗∗	0.894∗∗	0.883∗∗
AVE (average variance extracted)	0.699∗∗	0.657∗∗	0.741∗∗	0.653∗∗
$\sqrt{AVE}$	0.836∗∗	0.810∗∗	0.861∗∗	0.808∗∗

∗∗*p* ≤ .01.

### 4.6 Final hypotheses testing results

**Figure 1:**
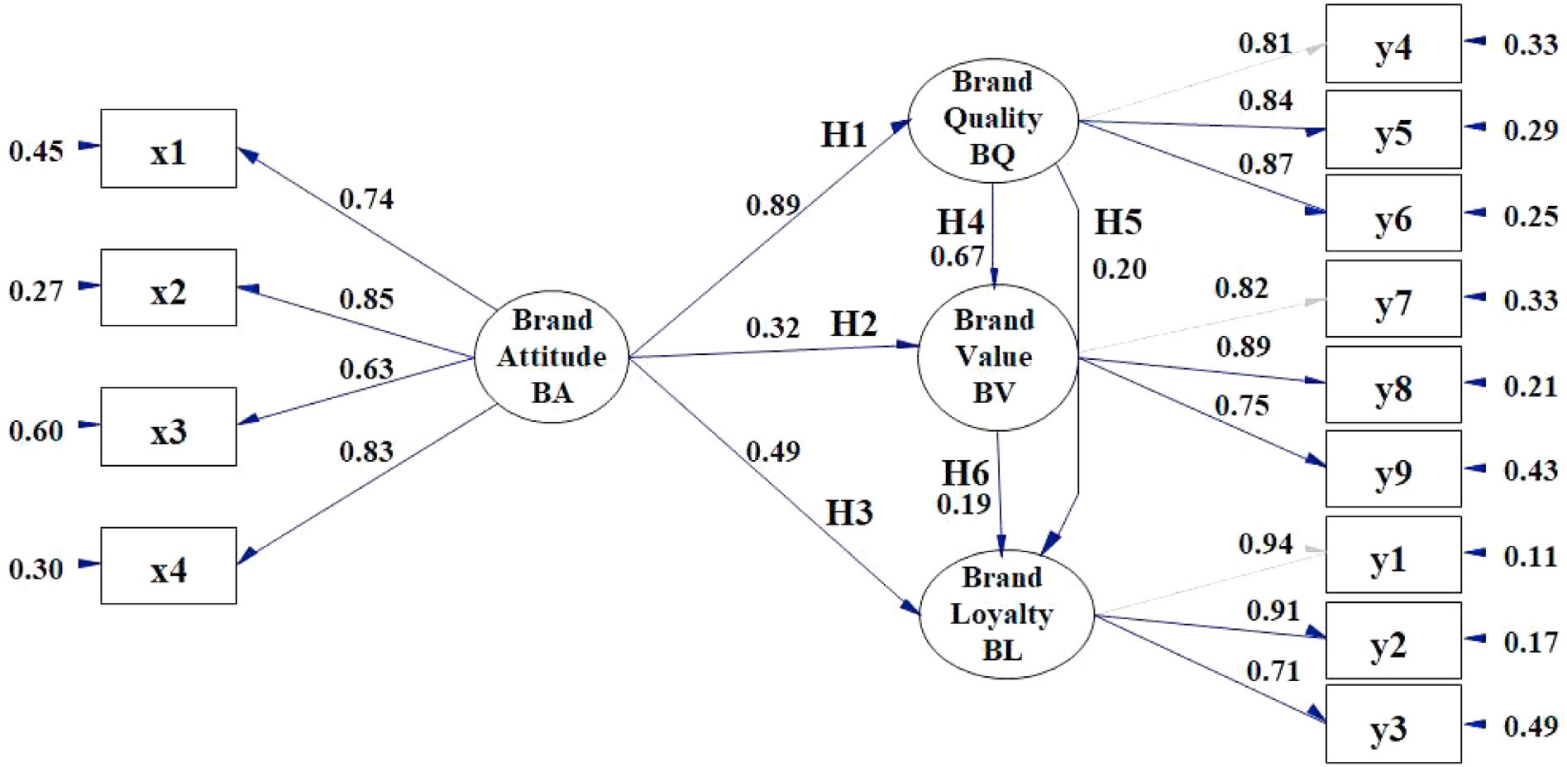
Final results of the SEM for Thai canned tuna BL. Chi-Square = 30.15, degrees of freedom df = 37, *p*-value = 0.78020, RMSEA = 0.000. Please see Table 1 for the observed variable (x1, y4, etc.) element descriptions.

The hypotheses testing results are shown in Table 5 as well as Figure 1. Therefore, of the six conceptualized hypotheses, four were validated whose results from weakest to strongest, were H2 (*r* = 0.32), H3 (*r* = 0.49), H4 (*r* = 0.67), and H1 (*r* = 0.89).

**Table 5:** Results of the hypotheses testing Thai canned tuna BL.

Hypotheses	*r*	t-value	Validity
Hypothesis 1: The presence of a consumer's brand attitude positively and directly affects their perception of brand quality.	0.89	16.44∗∗	valid
Hypothesis 2: The presence of a consumer's brand attitude positively and directly affects the brand's value.	0.32	3.39∗	valid
Hypothesis 3: The presence of a consumer's brand attitude positively and directly affects the consumer's brand loyalty.	0.49	3.53∗∗	valid
Hypothesis 4: Brand quality consumer perception positively and directly affects a brand's value.	0.67	6.76∗∗	valid
Hypothesis 5: Brand quality consumer perception positively and directly affects the consumer's brand loyalty.	0.20	0.83	Not valid
Hypothesis 6: A consumer's perception of brand value positively and directly affects the consumer's brand loyalty.	0.19	0.63	Not valid

∗ = *p* ≤ .05; ∗∗ = *p* ≤ .01, *r =* correlation coefficient.

## 5 Discussion

Results revealed that the R^2^ of 73% and the total effects of consumers' attitudes (BA) (labeling, brand confidence, packaging, and size & material) toward the five canned tuna brands were most important (0.85). This was followed by BQ (0.33), and BV (0.19) (Table 4). Further detail follows:

### 5.1 Brand attitude (BA) results

Results for the three hypotheses testing for BA was all positive and direct, with H1's relationship from BA to BQ strong with *r* = 0.89, the t-test value = 16.44, and *p* ≤ 0.01. The relationship from BA to BV was moderate with *r* = 0.32, the t-test value = 3.39, and *p* ≤ 0.05. Finally, H3's relationship from BA to BL was also moderate with *r* = 0.49, the t-test value = 3.53, and *p* ≤ 0.01.

Confirmation of a consumer's BA importance is easy to find in Thailand as ‘beauty’ is often judged to be a predominant component element in a consumer's rational for product selection (Chaipraditkul, 2013). In Thailand, the importance of brand labeling and packaging beauty cannot be understated, as aesthetics plays a major role in branding in Thai society. Therefore, aesthetics and symbols are an intrinsic part of a high-context Thai marketing culture (Andrews and Siengthai, 2009).

**Figure 2:**
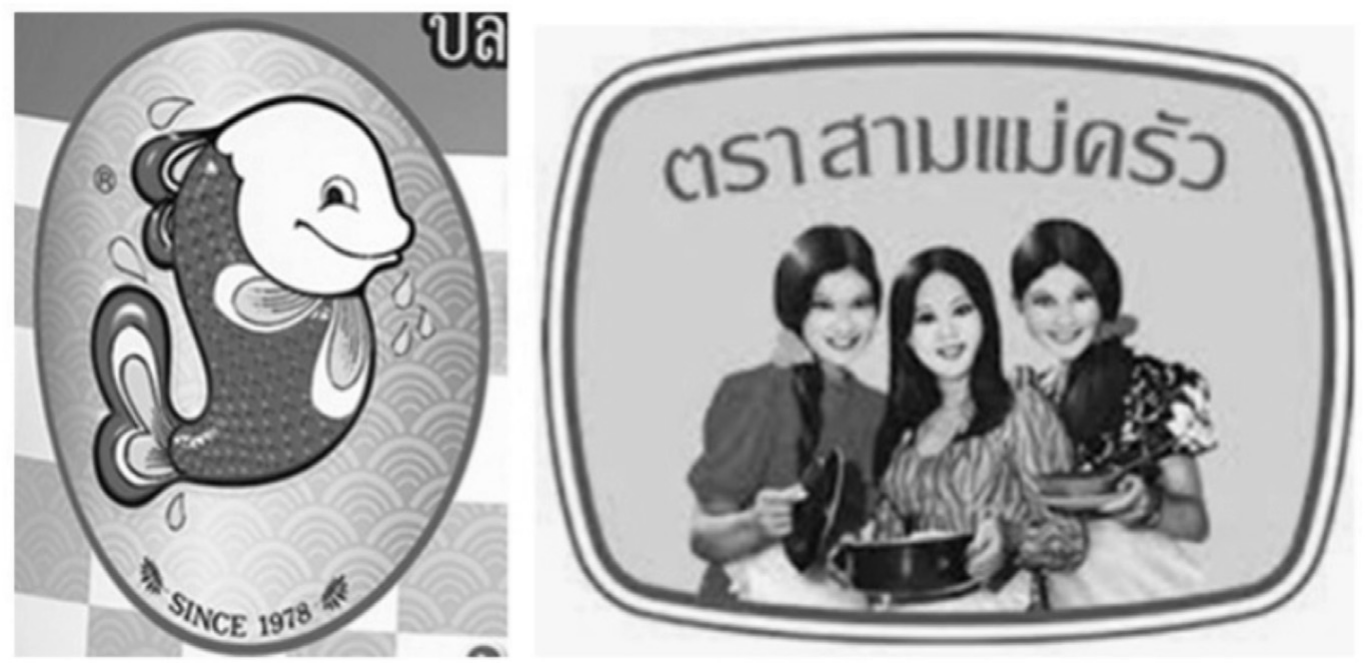
Pumpui canned tuna brand *Happy Fish* logo (left) and *Three Lady Cooks* brand logo (right).

Other evidence of a symbol's importance in Thai aesthetics comes from the *Superbrands Council of Thailand* which each year gives awards for the most creative and innovative brands. In recent years, the canned tuna brand Pumpui's happy fish logo on red packaging set it apart from its competitors and was an award recipient (Figure 2) (Andrews and Siengthai, 2009). Additionally, according to the ‘Three Lady Cooks/Royal Foods’ website, the *Three Lady Cooks* brand is also well known, and has also won awards as Thailand's ‘most recognized brand’.

Additional support for the importance of *attitude* in product decision making for canned tuna products can be found under the four 'packaging' items of the survey (Items 18–21). In it, item 21 was determined to be the most important, which is a can that has an easy to open design (pop-top) (Table 6). Research from Hana (2012) lends support to the importance of consumer attitude, as it was determined that once consumers have confidence in a firm and become attached to a certain brand, they are more likely to purchase it. Support for these results can also be found from research on Latin America canned tuna consumers. According to Chemerinski (2012), canned tuna is viewed as a versatile, healthy foodstuff and a 'must-have' in the house in Latin America.

**Table 6:** Questionnaire items concerning packaging attitudes.

Questionnaire Items for Packaging	mean	S.D.∗
I am satisfied with the various types of packaging used.	4.18	1.24
The can design is easy to store and appropriate.	4.49	1.11
The can design is interesting.	4.42	1.07
The folding cover design is easy to open.	4.52	1.12
Average	4.40	1.17

∗S.D. = standard deviation.

Support for H2 can be found in Iran where research concerning luxury items determined that brand attitude positively influences perceived value (Salehzadeh and Pool, 2017). Also, according to Zaeema and Hassan (2016), in the Maldives, demographic factors also influence canned tuna brand purchase decisions, as female consumers were more likely to purchase a canned tuna product than men. Also, cheaper canned tuna from Thailand is an ongoing competition concern for the Maldives' two local canneries. Moreover, from use of the standard Pearson's correlation coefficients strength interpretation and further analysis of other studies, we interpreted the study's results in the following manner:

### 5.2 Brand quality (BQ)Results

Results for the two hypotheses testing for BQ was positive and direct, with H4's relationship from BQ to BV strong with *r* = 0.67, the t-test value = 6.76, and *p* ≤ 0.01. However, the relationship from BQ to BL was determined to be not valid with *r* = 0.20 and the t-test value = 0.83. A follow-up analysis by consumer questioning seemed to indicate that there was a deeply entrenched identification with one of the surveyed brands, which was served at home by mother when the surveyed consumer was young (*Three Lady Cooks* brand). This is consistent with Italian consumer research concerning tuna products, in which it was concluded that consumers' ideas about food quality are complex, indeterminate, and uncertain (Conte et al., 2014).

### 5.3 Brand value (BV) results

The relationship between BV to BL in H6 was also unsupported with *r* = 0.19 and the t-test value = 0.63. The authors speculate that the reason for this is the consumers' response to items 40 and 41, in which the Thai consumers judge brand accessibility as more important than other concerns, such as price and quality (Table 7). Also, items 27 and 48 seem to imply that product quality and taste are low priorities when it comes to making a purchase decision. Could this be the reason (convenience is more important than price or quality) 56,000 convenience stores such as 7-Elevens (20,000 stores), Family Mart, and Lawson are such runaway successes in Japan (Obe, 2019). Moreover, in Thailand, there are over 10,268 7-Elevens as well (CPAll, 2020). Convenience seems to be paramount in importance in these workaholic societies.

**Table 7:** Questionnaire brand and quality comparisons.

Questionnaire Items for Branding	mean	S.D.∗
My brand is easy to find in most shops and stores.	4.63	1.16
My brand is always available.	4.60	1.18
I like my brand more than another brand.	4.03	1.39
My brand has a good quality.	3.95	1.25

∗S.D. = standard deviation.

### 5.4 Brand loyalty (BL) results

Multiple studies have stated the importance of the development and successful long-term maintenance of relationships with a customer (Baalbaki, 2012; Chow and Holden, 1997), especially when the pace of product change quickens (Rust et al., 2000).

This is supported by research from the UK concerning consumer's perceptions and preferences for tuna products, which determined that consumers from the middle to lower classes chose fish for its convenience (Nevin, 2003). Additionally, packaging beauty plays a key role to the Thai consumer if multiple product brands are an option. For the millennial generation, “if it was good enough for mother, it is good enough for me” attitude seems to also override other considerations such as price and quality.

Sustainability is also becoming an important component in canned tuna/tuna product branding and consumer selection, with various global organizations exerting great pressure and expense at judging which brands are *consumer worthy* (Greenpeace USA, 2016). Evidence for this comes from a Greenpeace study in which it was reported that only 55% of the 20 brands surveyed in USA supermarkets passed their canned tuna sustainability criteria (Thomson, 2017). Similar studies are also compiled for the Thai and Southeast Asian markets (Greenpeace USA, 2016). Another example of sustainability concerns is in Australia, with their major canned tuna brands. In 2018, a report from Havice and Campling (2018) named brands and companies for not supporting sustainable fishing practices.

There is no doubt that the most preferred canned tuna brand in Thailand is the *Three Lady Cooks* from Royal Foods (Table 8, Figure 2). Once again, a partial explanation could come from the longevity of the brand and the high possibility that ‘mom’ served it to the current consumer generations as early as 1973. Although *Pumpui*'s happy fish logo is cute, appealing, and award winning, it does not appear to be strong enough to compete with ‘mom’ and her generation's *Three Lady Cooks* product brand selection in Thailand. Further investigation of the *Pumpui* brand also discovered the lack of English language information and advertising, as it seems all marketing and advertising is focused only on the Thai consumer in the Thai language.

**Table 8:** Questionnaire brand and quality comparisons.

Preferred brand	Respondents	%
Three Lady Cooks (Royal Foods)	153	38.25
Select (Thai Union Frozen – TUF)∗	76	19.00
Roza (Hi-Q Food Products Co., Ltd.)	57	14.25
Super C-Chef (Sea Value Group)	57	14.25
Pumpui (Kuang Pei San Food Products Co.Ltd.)	57	14.25
Total	400	100.00

∗ The Select brand falls under the world's largest canned tuna company in the world; Thai Union Frozen – TUF (Greenpeace USA, 2016).

The results or data that support the conclusions shown directly or otherwise are available to the public in accordance with field standards.

## 6 Conclusion

Key takeaways from the study's research seem to indicate that canned tuna is viewed by most consumers as a *commodity*, with product availability and convenience being of the utmost importance in product brand selection. Moreover, seafood brand loyalty is a concept going back 100s of years which has involved not only companies, but international trading consortiums, and nation states. Furthermore, the practice of bringing tuna from the sea to the consumer has been a tale romanticized in all forms of art, with the tall tale of Pinocchio, to the art of Dali, to the film of Cousteau; with conflict at the center of each story. Today, that theme continues in the form of international quotas, trade wars, and labor and fishing fleet conflicts with various international organizations. Tuna, however, is critical to the world's food supply. As such, it must be studied and understood to be sustained.

## 7 Potential study limitations

The study is limited in that only Bangkok supermarket consumers who were Thais were selected for the survey. Additionally, there was no input into the study from Thailand's expat community or foreign tourists residing and shopping in the targeted areas. A similar survey in Cambodia or Vietnam would most probably have to use other regional or national brands. Additionally, a similar canned-tuna consumer study conducted outside Southeast Asia will most probably conclude differently. It might also be interesting to add the aspects of religious preference in a future study as well as when the product is consumed (e.g. Friday at work or at home for dinner). From the research, we also determined that there was an ongoing shift in consumer perceptions and concerns about canned tuna products. Follow-up research might focus on these changes including fleet and national labor practices, catch safety, sustainability, and perceptions about national quotas.

## 8 Contribution to the research

This very recent study addresses the importance of an economical wellspring of protein and potential Omega-3 food source, Tuna. However, brand research is very limited for canned tuna, especially when it comes to product packaging cues, and how packaging affects a consumer's decision-making process. This paper, therefore, unveils some of these mysteries in Thailand, and finds that family tradition (Mom) plays a critical role in a younger generation's canned tuna purchase decision making. Although quality and price are most often thought of as crucial elements in purchase decision making, this paper discoveries this is not the case for Thai consumers and canned tuna. We suspect similar surveys in other Asia nations such as Japan might yield similar results.

The paper also travels the world, investigating numerous studies concerning the history, harvesting, and branding of tuna as a food source. As such, the paper highlights where tuna is harvested, conflict is not far off. Very recent support for this statement can be found in a small group of eight Pacific nations, in which their Parties to the Nauru Agreement (PNA) is a declaration of national rights (and their fight for) to their greatest natural resource, tuna. We believe this paper highlights these global concerns and conflicts.

## Declarations

### Author contribution statement

Pichit Chuenban: Conceived and designed the experiments; Performed the experiments; Analyzed and interpreted the data; Contributed reagents, materials, analysis tools or data; Wrote the paper.

Puris Sornsaruht: Conceived and designed the experiments; Analyzed and interpreted the data; Contributed reagents, materials, analysis tools or data.

Paitoon Pimdee: Analyzed and interpreted the data; Contributed reagents, materials, analysis tools or data.

### Funding statement

This research did not receive any specific grant from funding agencies in the public, commercial, or not-for-profit sectors.

### Data availability statement

Data included in article/supplementary material/referenced in article.

### Declaration of interests statement

The authors declare no conflict of interest.

### Additional information

No additional information is available for this paper.
